# Sustainable slag-based geopolymers incorporating styrofoam and Moringa leaves ash for mechanical enhancement and crystal violet adsorption

**DOI:** 10.1038/s41598-025-20119-1

**Published:** 2025-10-07

**Authors:** Soher A. Hashish, Essam A. Kishar, Doaa A. Ahmed, Sheren M. Ragei, Aya Allah M. Ebrahim

**Affiliations:** Chemistry Department, Faculty of Women for Arts, Science and Education, Ain Shams University, Cairo, 11757 Egypt

**Keywords:** Slag geopolymer, Styrofoam, Moringa leaves ash, Crystal violet, Batch adsorption, Environmental sciences, Materials science

## Abstract

Synthetic dye contamination poses a significant threat to water resources, necessitating the development of efficient removal technologies. This study introduces a novel organic-inorganic modified geopolymer for effective Crystal violet (CV) dye extraction from aqueous solutions. Additionally, it addresses waste management challenges by incorporating industrial (Slag, Styrofoam) and agricultural (Moringa leaves ash) waste into geopolymer cement. Slag-based geopolymer composites were synthesized with varying concentrations (1, 3, and 6 wt%) of Styrofoam (SSF1, SSF3, SSF6) and moringa leaves ash (SMA1, SMA3, SMA6). The mechanical properties of these composites were assessed over a curing period of up to 180 days in 100% humidity. While the control geopolymer sample (S) demonstrated superior compressive strength of 58.9 MPa after 180 days of curing, the modified geopolymer composites exhibited notable mechanical stability, with SSF1 and SMA1 achieving strengths of 52.8 MPa and 47.5 MPa, respectively. Furthermore, the adsorption performance of the modified geopolymer composites toward CV was evaluated as an additional environmental application. Notably, all modified geopolymers surpassed the control in CV removal, with SSF6 exhibiting an outstanding adsorption capacity of 434.78 mg/g. These findings demonstrate the potential of modified geopolymer composites as a promising, sustainable, and high-performance material for wastewater treatment applications.

## Introduction

The demand for more environmentally friendly building materials, geopolymers have gained attention as a strong substitute for conventional Portland cement. While geopolymers and PC may share comparable qualities, geopolymers are particularly promising since they are derived from materials that require less energy and emit less CO_2_, like calcined clay, or industrial byproducts such as fly ash and slag with alkali activators^1^. Slag, a highly reactive aluminosilicate substance, is created during the iron production from iron ore. It has been used expansively in the creation of geopolymers^2^. Beyond construction, we’re urgently tackling the massive problem of Styrofoam waste. Styrofoam is a synthetic polymer made from monomers of the aromatic hydrocarbon styrene^3^. Its production reaches several million tons per year^4^. Millions of tons of this synthetic material are produced yearly, taking over 500 years to decompose and releases large amounts of ozone into the atmosphere during manufacture^5^. Innovative recycling efforts are exploring its potential as a a binder^6,7^, an adsorbent^8^or even as aggregate in lightweight concrete, all aiming to reduce its environmental footprint^9^.

Nature also offers solutions, notably through Moringa oleifera, a fast-growing, drought-resistant tree^10^. Its seed powder shows promise in creating sustainable concrete, and its leaves, particularly when turned into biochar, are proving effective at purifying wastewater^11–13^, including the removal of harmful dyes from textile effluent^14^.

Over the past few decades, rapid industrialization has turned water pollution into a serious environmental issue concern. This brings us to a significant environmental challenge: water pollution from industrial dyes like Crystal Violet (CV). These dyes are not only persistent in water but also harmful to aquatic life by blocking sunlight and hindering photosynthesis. Furthermore, CV exposure poses serious risks to human health, such as respiratory issues and skin irritation, and potential carcinogenic effects. Addressing the pollution caused by CV requires effective remediation strategies. Recent studies have explored various methods, including biodegradation using specific bacterial strains capable of breaking down CV into less toxic compounds and the adsorption method using eco-friendly adsorbents^15–18^.

While various methods exist for cleaning wastewater^19,20^, adsorption is highly regarded for its effectiveness, affordability, and simplicity^21^. Lately, researchers have focused on developing adsorbents that are environmentally friendly, inexpensive, and highly effective^22^. One promising alternative for this purpose is a geopolymer^22^. Recent breakthroughs in developing microporous and mesoporous geopolymers have greatly improved their ability to act as adsorbents, offering an eco-friendly path toward cleaner water and a healthier planet^23,24^ Both metakaolin geopolymer and moringa slag based geopolymers, demonstrate significant ability to adsorb dye from aqueous solutions due to their porous structure and surface chemistry^25,26^. Modifications such as incorporating waste charcoal powder enhance adsorption capacity by increasing surface area and functional group density, achieving ultrahigh adsorption capacities exceeding 2800 mg/g in some composites^27^. These properties make geopolymers promising, efficient adsorbents for CV removal in wastewater treatment, although further research is needed to improve reusability and practical application^28^. However, no research has explored the combined effects of agricultural waste, specifically Moringa leaves, and organic industrial waste, such as Styrofoam, in slag-based geopolymer cement for environmental applications.

This research investigates a sustainable attempt to waste valorization by using industrial (Slag, Styrofoam) and agricultural (Moringa leaves ash) byproducts. The study utilizes Moringa leaf ash (MLA), chosen for its high silica and alumina content, which enhances structural integrity and reactivity, and Styrofoam (SF) as a pore-forming agent. This method addresses environmental concerns related to waste disposal and promotes sustainability in construction. The study highlights the effects of different amounts of SF and MLA on the mechanical performance of slag-based geopolymer composites. Furthermore, the study assesses the adsorption capacity of these composites in treating industrial effluent contaminated with crystal violet (CV) dye. The results demonstrate the potential of these novel organic-inorganic slag-based geopolymers for water treatment, highlighting their dual function in waste valorization and environmental remediation.

The findings indicate that Styrofoam and moringa leaf ash can be used to produce sustainable geopolymer composites with improved mechanical and adsorptive properties. Future research should focus on their applications in construction and environmental remediation to further improve the long-term sustainability of these composites.

## Materials and methodology

### Materials

**Fig. 1:**
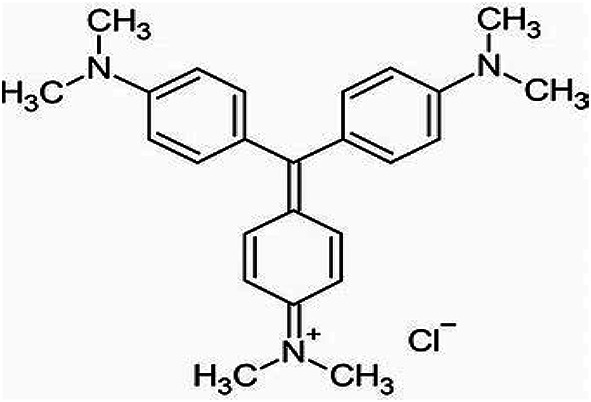
Structure of crystal violet.

Ground granulated blast furnace slag (GGBFS) with particles size smaller than 100 μm was provided from Nile Oversees Company (Cairo, Egypt). Styrofoam was collected from packaging materials. The collected Styrofoam was cleaned and sorted to remove contaminants. The Moringa Oleifera leaves were gathered from a tree in the El Haden Area (El Behera, Egypt). Sodium hydroxide (NaOH) flakes and liquid sodium silicate (LSS) were supplied by EL Goumhouria Chemical Company (Cairo, Egypt), and the Silica Egypt Company (Alexandria, Egypt). The silica ratio of SiO_2_/Na_2_O equals 2.80 and the composition of liquid sodium silicate is 11.7 wt% Na_2_O, 32.8 wt% SiO_2_, and 55.5 wt% H_2_O. Chemically pure Crystal violet (molar weight = 408.0 g mol^– 1^, λ-max = 590 nm, purity of 99.0%) was supplied from Prolabo company (France) (Fig. 1). The entire experiment proceeded with distilled water. The Blaine surface area of ground granulated blast furnace slag is 4700 × 50 cm^2^ per gram.

**Fig. 2:**
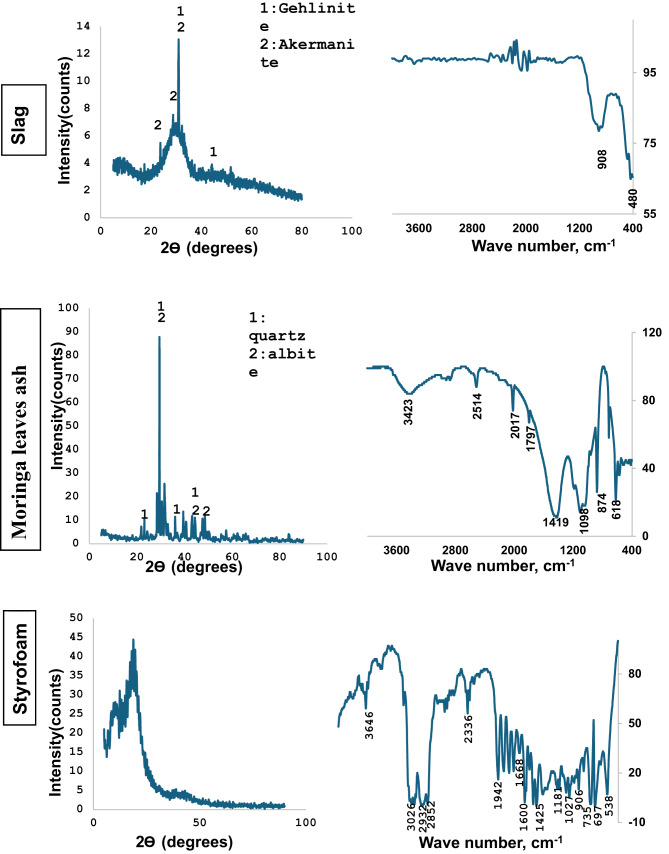
The XRD and FTIR of raw materials.

Figure 2 displays the XRD and FTIR spectra of the raw materials. The XRD spectrum of GGBFS indicates broad peaks at 2θ = 25–35, which are owing to the glassy phase’s high active silica content. Weak crystalline peaks at 2θ = 29.12 and 31.25 confirm the presence of akermanite and gehlinite. The FTIR spectrum of GGBFS displays typical characteristics of slag, with bands at 908 cm^−1^ related to Si–O–T (T = Si or Al) asymmetric stretching vibration and 450–470 cm^− 1^ indicating Si–O–Si bending vibration mode.

Moringa leaves ash (MLA) exhibit quartz and albite as the main phases in the XRD spectrum and shows characteristic bands in the FTIR spectrum at 3431 cm^−1^ (linked to the presence of –OH group), 1098 cm^−1^ (linked to the vibrations of asymmetric Si-O-Si stretch), 874 cm^−1^ (vibrations of symmetrical stretching Si-O-Al), and 619 cm^−1^ (characteristic of crystalline cristobalite).

Styrofoam has an amorphous nature with a wide XRD signal around 2θ = 20°^29^. Its FTIR spectrum shows intense bands at 3026 cm^− 1^ (indicating the presence of aromatic C-H), 2932 and 2852 cm^−1^ (representing aliphatic C-H), and 1600 and 1425 cm^−1^ (linked to C = C stretching). Additionally, there is a group of peaks below 1000 cm^− 1^ (735, 697, and 538) due to the aromatic ring’s C-H bending modes.

### Geopolymer characterisation and methodology

#### Geopolymer composite preparation

##### Preparation of MLA

The leaves of Moringa oleifera are collected from agricultural areas. To remove dirt and contaminants, the leaves were washed with clean water, dried for 24 h at 100 °C, and then allowed to cool to room temperature. The dried leaves were crushed into small pieces using a mechanical crusher, followed by ultrafine grinding for a finer texture. The final powder was heated in an oven at 600 °C for 2 h^30,31^ and sifted via a 100-mesh sieve to achieve a consistent particle size. The powdered moringa leaves were stored away from direct sunlight in a cool, dry place.

##### Preparation of alkaline activator

The alkali, an activator solution, was made by mixing sodium hydroxide (10 M) and sodium silicate in a weight ratio of 2.5 and letting to cool for 24 h.

##### Synthesis of geopolymer

**Fig. 3:**
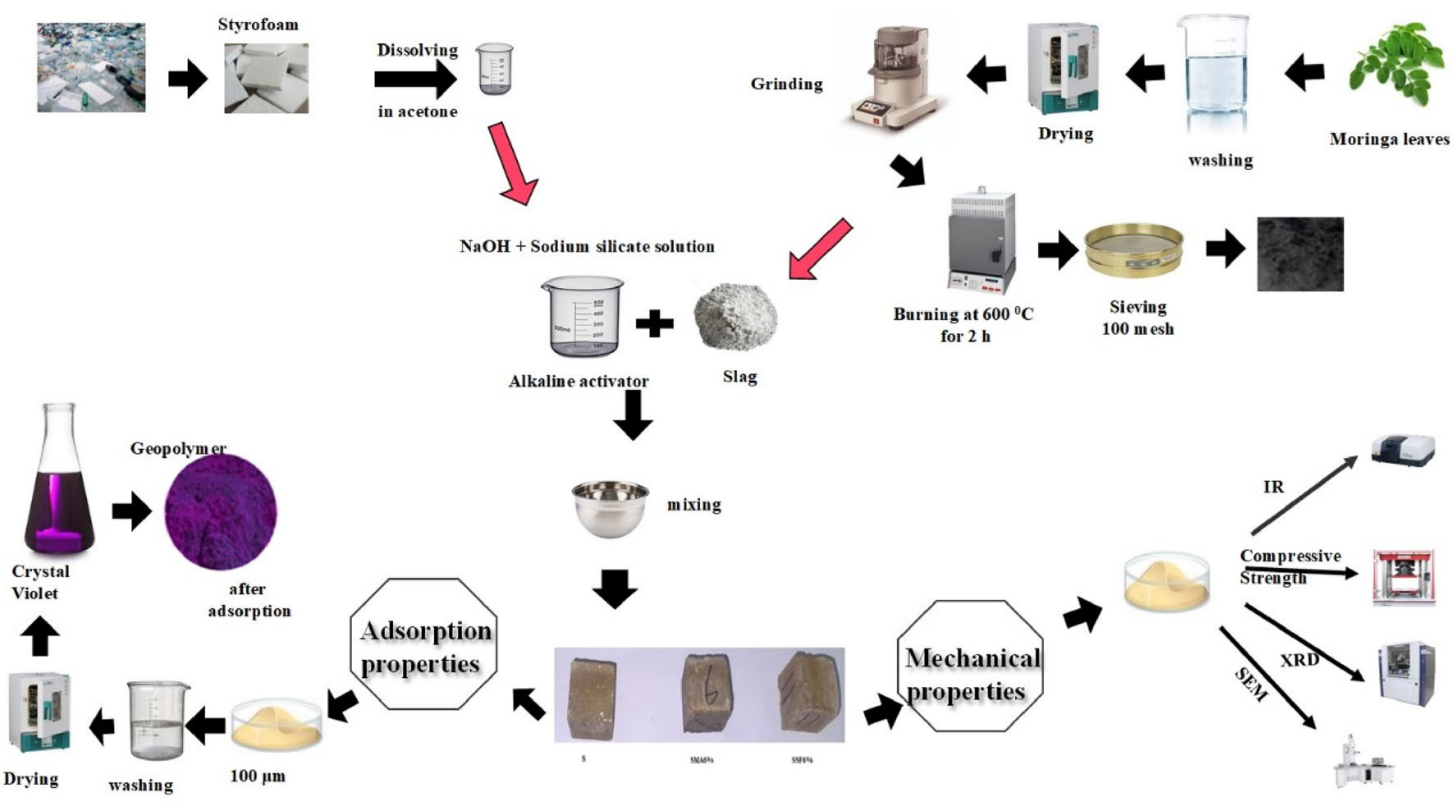
Schematic diagram of the experimental process.

Firstly, Styrofoam was dissolved in acetone to obtain a smooth paste^7^. To the dried mixtures, an alkaline activator solution was added and mixed for 15 min. Table 1 displays the constituents of the geopolymer composites. In order to enhance the mechanical properties in addition to adsorption characteristic, the maximum percentage chosen is 6% for both MLA and Styrofoam^32,33^ After mixing, the sample pastes were poured into molds (2.5 cm x 2.5 cm x 2.5 cm), then cured for 24 h at 60 °C. Following the curing period, the geopolymer cubes were removed from the molds and stored at 100% humidity. XRF was used to analyze the chemical oxide contents of ground granulated blast furnace slag and Moringa Oleifera leaves ash (MLA) in Table 2. The schematic diagram given in Fig. 3 summarizes a description of the preparation of geopolymer, characterization and adsorption test.

**Table 1:** The composition of the geopolymer paste mixes.

Mix code	S	SMA1	SMA3	SMA6	SSF1	SSF3	SSF6
Wt. % of slag (GGBFS)	100%	99%	97%	94%	99%	97%	94%
Wt.% of Moringa leaves ash (MLA)	–	1%	3%	6%	–	–	–
Wt.% of styrofoam (SF)	–	–	–	–	1%	3%	6%

**Table 2:** Chemical oxide compositions (mass %).

Chemical composition %											
	SiO_2_	Fe_2_O_3_	CaO	Al_2_O_3_	MgO	SO_3_	Na_2_O	K_2_O	L.O.I	Cl^−^	H_2_O
Slag	32.86	1.14	42.56	7.02	11.58	2.50	0.29	0.15	0.93	0	0
MLA	38.16	14.90	18.10	17.20	4.76	1.71	1.18	1.84	0.24	0.06	0

#### Characterization of geopolymer

To assess each paste’s compressive strength, a D550-control type machine from Milan, Italy was used. An alcohol/acetone (1:1) solution was used to end the hydration process of the crushed cubic specimens and then dried for 24 h at 50 °C. The total porosity of each hardened paste was assessed by Brunauer-Emmett-Teller (BET) surface area analyzer using the Quanta Chrome model of NOVA touch 2LX, Milano-Italy machine. High-resolution scanning electron microscopy (ESEM/Mapping: FEI, model Quanta FEG 250), infrared spectrophotometer (PerkinElmer 1430 infrared spectrophotometer, USA), and X-ray diffraction (Bruker D8 Discover diffractometer, Germany) were used to examine the phase composition, functional groups, microstructure, and morphological development of the hydration products. The textural characteristics were recognized using adsorption-desorption of the N_2_ gas technique at 77^o^ K using (MINI X, MICROTRAC). To calculate the average pore diameter (nm), specific surface area (m^2^/g), and total pore volume (cm^3^/g), Brunauer-Emmett-Teller (BET) and Barrett-Joyner-Halenda (BJH) models were employed.

#### Adsorption test

The removal efficiency and adsorption capacity of geopolymer composite mixes were studied using batch adsorption tests with varying crystal violet dye (CV) initial dye concentrations (10–120 mg L^− 1^), adsorbent dosages (0.01–0.2 g L^− 1^), contact time (30–360 min), and pH (2–8) at room temperature (25°c). The experiments were accomplished in batches using 50 mL dye solution in a shaker. A UV/visible spectrophotometer was used to quantify the concentration of the supernatant at λ-max = 590 nm. Equation (1) calculates the dye removal effectiveness (%), while Eqs. (2) and (3)^34^ were used to quantify the adsorbed amount at equilibrium (qe, mg g^–1^), as well as the adsorbed quantity at a certain time t (qt, mg g^–1^).

1 $$\:\:\:\:\:\:\:\:\:\:\:\:\:\:\:\:\:\:\:\:\:\:\:\:\:\:\:\:\:\:\:\:\:\:\:\:\:\:\:\:\:\:\:\:\:\:\:\:\text{R}\:\left(\text{\%}\right)=\frac{\left({C}_{o}\:-{C}_{t}\right)}{{C}_{o}}\times\:100$$

2 $$\:\:\:\:\:\:\:\:\:\:\:\:\:\:\:\:\:\:\:\:\:\:\:\:\:\:\:\:\:\:\:\:\:\:\:\:\:\:\:\:\:\:\:\:\:\:\:\:\text{q}\text{e}=\frac{\left({c}_{o}-{c}_{e}\right)v}{m}$$

3 $$\:\text{q}\text{t}=\frac{({C}_{o}\:-{C}_{t})\text{v}}{m}$$

Ce (mg L^− 1^) is the concentration of adsorbate by an adsorbent at equilibrium, V is the volume of solution (L), m is the amount of adsorbent (g), and C_0_ (mg L^− 1^) is the initial concentration of dye and C_t_ (mg L^− 1^) concentration of the dye at time t.

#### Isotherm models

The equilibrium data obtained for CV on various prepared geopolymer mixes were modeled applying the Freundlich and Langmuir models. The linearized forms of the two isotherms are^34^:

4 $$\:\frac{{c}_{e}}{{q}_{e}}=\frac{{c}_{e}}{{q}_{m}}+\frac{1}{{k}_{1}{q}_{m}}$$

5 $$\:\text{l}\text{n}{q}_{e}=\text{l}\text{n}{k}_{f}+\frac{1}{n}\text{l}\text{n}{c}_{e}$$

The equilibrium concentration is C_e_ (mg/L), while the amount of dye adsorbed at equilibrium is q_e_ (mg/g). The Langmuir constant is k_l_ (L/mg) while the monolayer adsorption capacity is q_m_ (mg/g). The maximum CV absorbed into the geopolymer (q_m_) and the Langmuir constant k_l_ was derived from the chart of C_e_/q_e_ against C_e_, as explained in Eq. (4). The Freundlich constants, k_f_ and n, represent the adsorption intensity and capacity, respectively. Similarly, the plot of ln(q_e_) vs. ln (C_e_), as shown in Eq. (5), can be utilized to obtain the Freundlich isotherm constants KF and n.

#### Kinetics models

Kinetic models are used in adsorption to explain how quickly adsorbents—such as particles, ions, or molecules—stick to an adsorbent’s surface. The kinetics data produced on the adsorption investigation for the influence of time were examined in our investigation using pseudo-second-order model (Eq. 6), and the pseudo-first-order model (Eq. 7)^34^.

6 $$\:\:\:\:\:\:\:\:\:\:\:\:\:\:\:\:\:\:\:\:\:\:\:\:\:\:\:\:\:\:\:\:\:\text{l}\text{n}\left({q}_{e}-{q}_{t}\right)=\text{l}\text{n}\:{q}_{e}-\frac{{k}_{1}}{2.303\:}\text{t}$$

7 $$\:\:\:\:\:\:\:\:\:\:\:\:\:\:\:\:\:\:\:\:\:\:\:\:\:\:\:\:\:\:\:\:\:\frac{t}{{q}_{t}}=\:\frac{1}{{k}_{2}{q}_{e}^{2}}+\:\frac{t}{{q}_{e}}$$

The amount of CV adsorbed at equilibrium is referred to as qe (mg/g) and at times t by qt (mg/g).

The pseudo-first-order adsorption rate constant is k_1_ (min ^−1^) and the pseudo-second-order one is k_2_ (g mg ^−1^ min ^−1^).

## Results and discussion

### Mechanical characteristics

#### Compressive strength and total porosity%

**Fig. 4:**
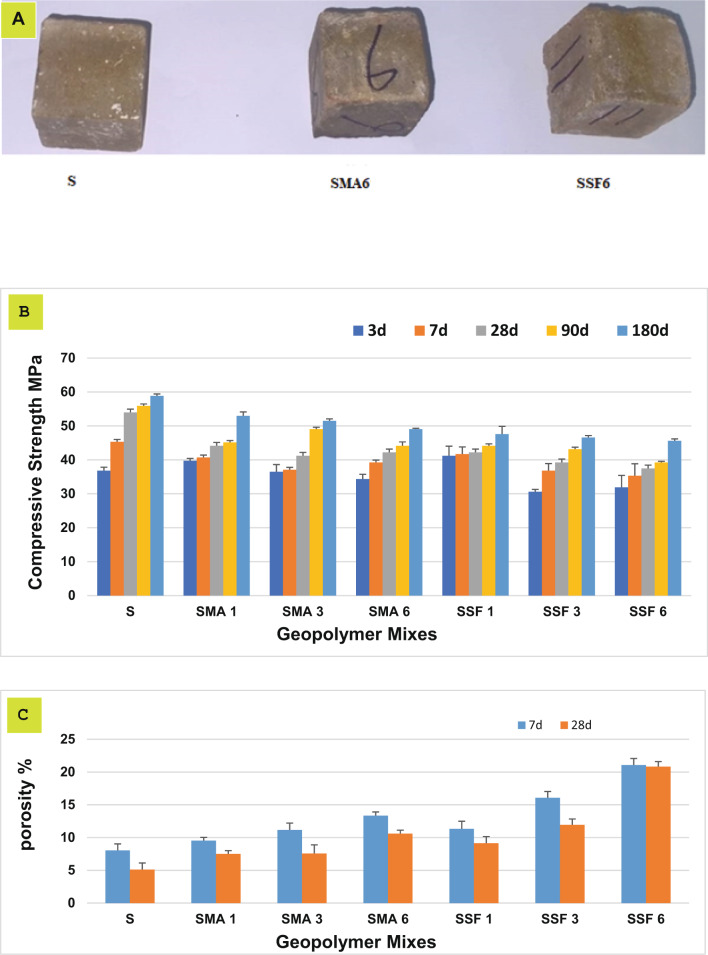
(**A**) Geopolymeric cubes of three different compositions, (**B**) The values of compressive strength and (**C**) total Porosity % for the geopolymer at different ages.

The compressive strengths of the reference specimen and the geopolymeric composites containing MLA and Styrofoam up to 180 days are shown in Fig. 4. It is clear that the compressive strength rises with time due to the development in the geopolymerization process, resulting in the development of additional strength-giving phases in the existing open pores, including calcium aluminate hydrate (CAH), calcium silicate hydrate (C-S-H), calcium alumino silicate hydrate (C-A-S-H), and sodium ammonium silicate hydrate (N-A-S-H) ^35^. By decreasing the porosity, the strength-giving phases create a compact matrix composite with superior mechanical properties as seen in Fig. 4. The mixes containing MLA gave lower compressive strength than the reference mix; this is properly due to the dilution of slag with less active pozzolanic material (MLA) and the lower dissolution of the silicate crystals (quartz and albite) encompassing the leaves’ ash because of their higher stability^36^. Furthermore, the MLA may cover the slag particles, preventing the slag from geopolymerizing^35^. Mixes with Styrofoam show the lowest compressive strength and the highest porosity after 28 days. The findings are probably due to the deficiency of coherence between the Styrofoam fiber and the geopolymer matrix^37,38^. So, incorporating MLA and SSF reduces the compressive strength and increases total porosity. Mix SMA6 and SSF6 will be further investigated to study their adsorption characteristics.

#### FTIR spectrum

**Fig. 5:**
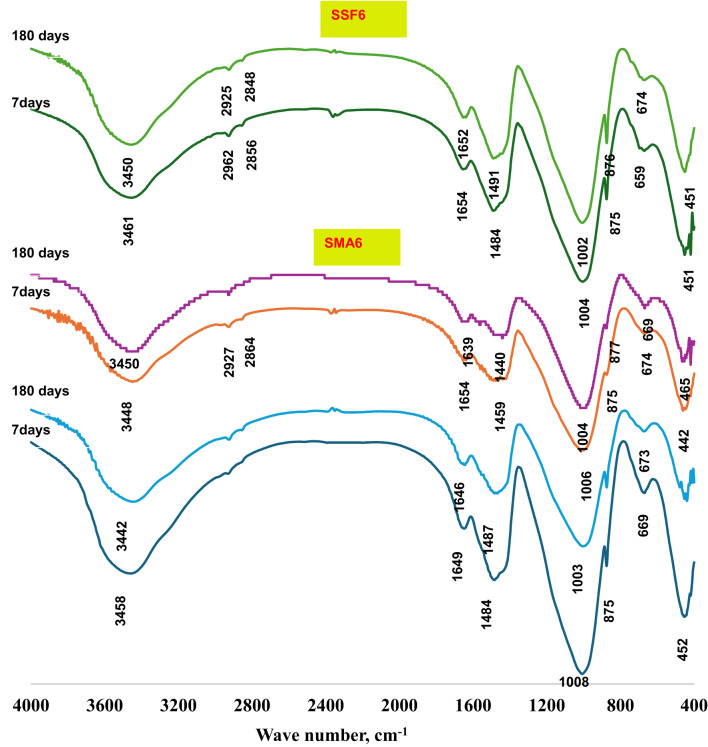
FTIR-spectra of the geopolymer at 7 days and 180 days.

The FT-IR spectra of the reference geopolymer paste (S) and the geopolymeric composite mixes (SMA6 and SSF6) at 7 and 180 days of hydration are shown in Fig. 5. All the samples were found to have seven transmittance bands at varying vibrational energies (cm^−1^). Wide bands were found within the range of 3440–3460 cm^−1^, that matches the symmetric stretching vibrations of the hydroxyl group^39^. H–O–H bending vibrations or O–H bending vibrations are accountable for the bands observed around 1650 cm^−1^^40^. The asymmetric and symmetric stretching vibrations of -CH_2_ originating from the ash and Styrofoam of moringa leaves are linked to the two distinctive bands that emerged between 2920 and 2850 cm^−1^^41^. The bands at 1000–950 cm^–1^ range were connected to Si–O–T asymmetric stretching vibration (where T is either Si or Al), which shifted to a lower wavenumber after 180 days. The bands around 875 cm⁻¹ are frequently linked to the bending vibrations of the Si-O-Al bond in the geopolymer network. This is probably owing to the dissolution of active silica and the creation of more intricate and stable aluminosilicate networks, like those in C-S-H and C-A-S-H gels^42^.The peaks at (659–674) cm^−1^ relate to the symmetric Si–O–Si (Al) bridges’ stretching vibrations in cyclo-silicates^43^, However, the Si-O-Si bending vibration mode at approximately (442–465) cm^–1^ was related to unreacted quartz^44^. During the carbonation process of Ca (OH)_2_, the C-O stretching vibration in CO3^−2^ groups forms the bands at 1420–1450 cm^−1^. The band at 875 cm^−1^ is slightly shifted because of the carbonation process that alters the chemical environment of the aluminosilicate network. From Fig. 5. It can be observed that mix SSF gives a more intense band at 1459 cm^−1^ after 180 days of hydration, which is probably because of its pores structure. While MLA shows a reduction in that peak, maybe due to the creation of more hydration products in the pores, which lowers the carbonation process.

#### X-ray diffraction

**Fig. 6:**
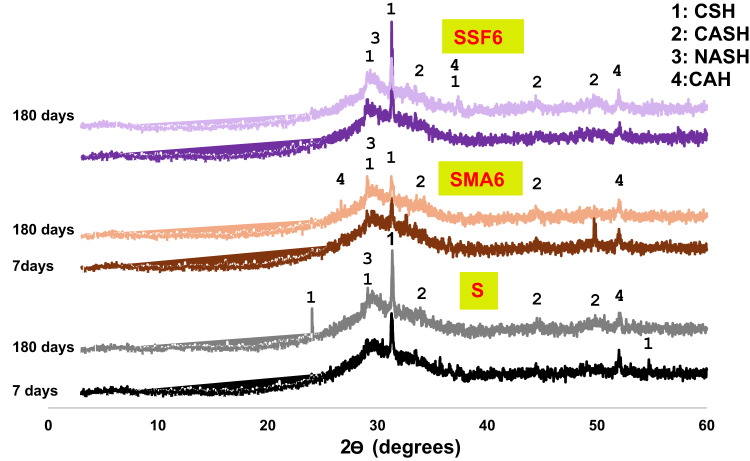
XRD of the geopolymer at 7 and 180 days.

It is essential to understand the phases that result from geopolymerization, as they influence the adsorption and mechanical properties of geopolymeric composites. Figure 6 shows the phase composition of the synthetic geopolymer composites (S, SMA6, and SSF6). Different phases were identified within the reference and geopolymer composite’s structure, aligned with the full dissolution of akermanite and gehlenite crystals presented in the pure GGBFS, such as sodium aluminosilicate hydrates (NASH) with known broadness between 22 and 35^◦^ because of the amorphous character of its chains, calcium aluminosilicate hydrates (CASH) at 2θ = 34, 44 and 52^◦^, calcium silicate hydrates (CSHs) at 2θ = 29^◦^ and calcium alumina hydrates (CAH) at 2θ = 37 and 51^[◦34^. Alkaline activation causes the precursor phases in the current geopolymer samples to dissolve significantly afterward polycondensation results in a more uniform aluminosilicate matrix, as indicated by the broadening or disappearance of the crystalline peaks (XRD of raw materials in Fig. 2). Early on, an amorphous hump of unreacted GGBFS was seen approximately at 30^o^, which corresponds to the C-S-H peak at 180 days. The intensity of the amorphous CSH hump becomes more pronounced over time, suggesting a more developed and homogeneous geopolymeric gel. Moreover, The NASH and CASH peaks become more prominent at 180 days. Generally, the increase in the amorphous phases and the reduction in unreacted precursor materials correlate with an increase in compressive strength. Furthermore, both SMA and SSF show a decrease in intensity of the hydration product characteristic peaks (CSH, CASH), indicating that the slag particles may be coated with MLA and Styrofoam, which would prevent the slag from geopolymerizing. This was coherent with the results of compressive strength.

#### SEM analysis

**Fig. 7:**
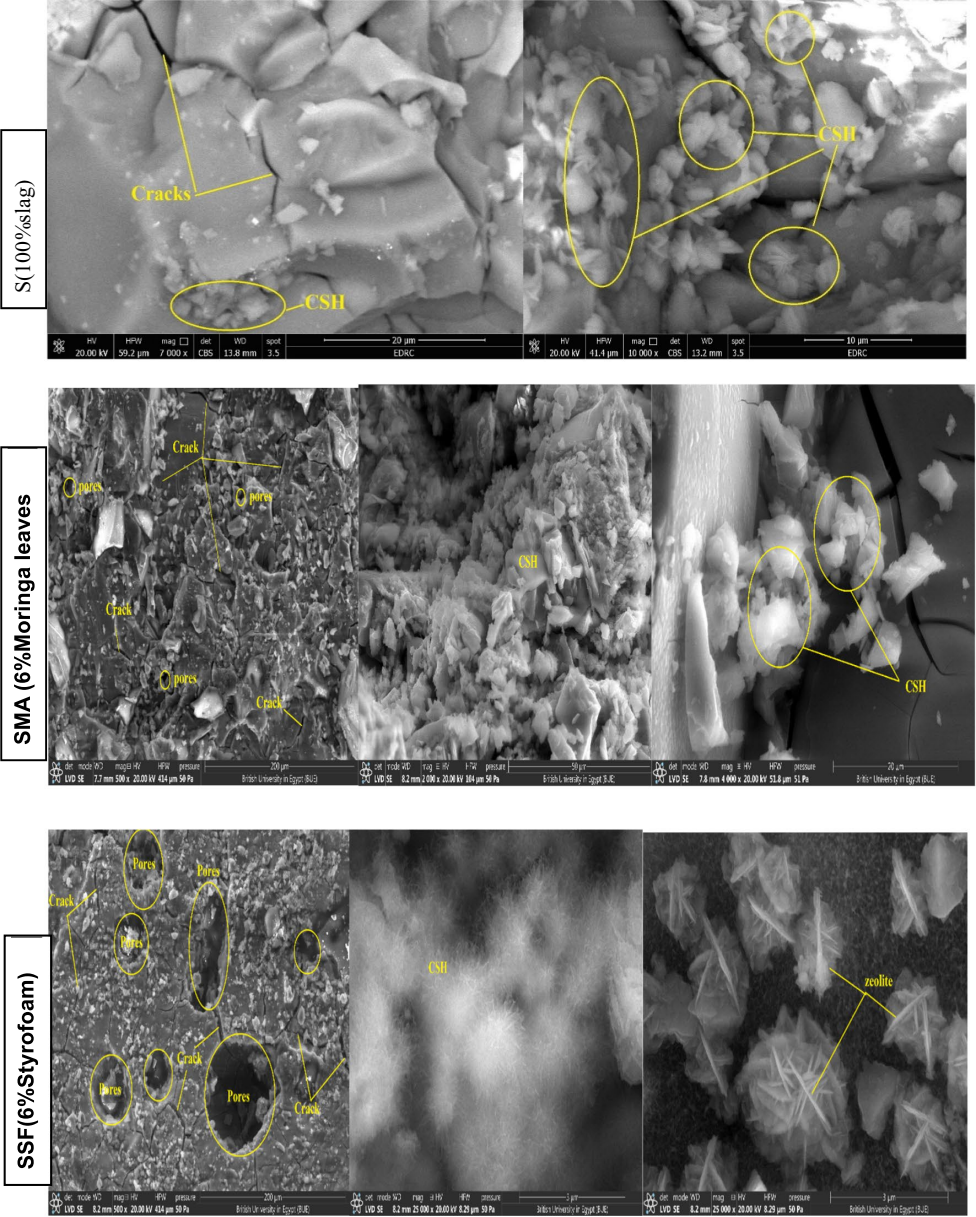
SEM micrographs of hardened geopolymer after 7 days of hydration at different magnifications.

In general, the microstructure of the geopolymeric matrix and the shape of the created binding phases have a major impact on the mechanical performance. The SEM micrographic images of the S, SMA6, and SSF6 after 7 days of hydration are demonstrated in Fig. 7. The presence of crosslinked fibers C-S-H is visible in micrographic pictures, which have greater filling ability, providing evidence for the development in the compressive-strength value and presenting a much denser matrix^45^. In mix S, a more uniform and smoother surface is a result of the particles’ denser packing, in particular. However, there is a different surface characteristic, with large interparticle gaps and uneven surfaces, in mix SMA6% and SSF6%. According to these findings, adding MLA and Styrofoam to the geopolymer matrix increases its porosity and surface roughness. Greater surface roughness and porosity both lead to a larger surface area. The BET study provides more evidence for these structural alterations^46^.

Moreover, SEM analysis of the samples revealed the emergence of several microcracks on the specimens’ surfaces, and SSF shows more cracks with a larger pore size (Table 3). This cracking is primarily caused by uneven shrinkage within the geopolymer matrix or by fast water loss via evaporation during the hardening phase^47,48^. This observation was consistent with the XRD and mechanical properties results, which confirm the lower mechanical properties of SMA6 and SSF6 than the reference mix (S). Furthermore, SSF6 shows the creation of interweaved fibers from C-S-H and a zeolite structure shaped like a flower. These findings are allied to the results reported in a recent study^49^.

**Table 3:** The main pore structure analysis characteristics parameter.

Adsorbent	SBET, m^2^.g^− 1^	Pore volume, cc.g^− 1^	Average pore radius, nm
S	11.27	0.03	6.02
SMA6	17.52	0.05	5.76
SSF6	53.18	0.08	3.00

#### Texture characteristics of the prepared geopolymers

**Fig. 8:**
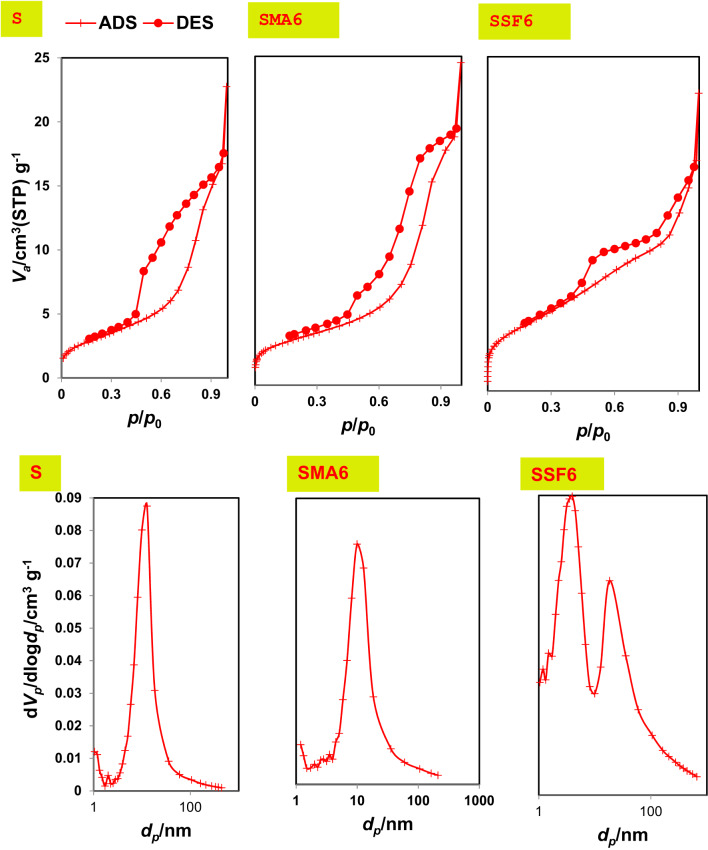
N_2_-adsorption/desorption isotherm and BJH-pore size distribution for all mixes, respectively.

The texture characteristics greatly help us explain the adsorption performance of the prepared geopolymers, using the N_2_-adsorption/desorption technique. BET models were used to determine important parameters like average pore diameter, specific surface area, and total pore volume. The BJH model was used to determine the types of pores (micro, meso, or macro). Moreover, isotherm types of geopolymer samples were categorized using IUPAC guidelines to detect the adsorption performance of the prepared geopolymeric composites^50^. The BET pore diameter, pore volume, and surface area are shown in Table 3. According to the data, the control mix’s (S) specific surface area, pore volume, and pore diameter are, respectively, 11.277 m²/g, 0.034 cm³/g, and 6.023 nm (Table 3). These findings suggest that Mix S possesses an H3 hysteresis loop and a mesoporous structure (Fig. 8). Following modification, the BET surface area, pore volume, and pore diameter for SMA6 and SSF6 (Table 3) changed to 17.524 m²/g, 0.050 cm³ /g, 5.759 nm, and 53.179 m²/g, 0.079 cm³ /g, 3.004 nm, respectively. Additionally, the SMA6 and SSF6 adsorbents fit more closely to a type IV isotherm than to a type VI isotherm and exhibit a mesoporous shape. The rougher structure seen in SEM micrographs and the larger surface area offer additional CV binding active sites, enhancing the material’s ability to adsorb and remove CV^51^.

### Adsorption investigations

#### Effect of pH

**Fig. 9:**
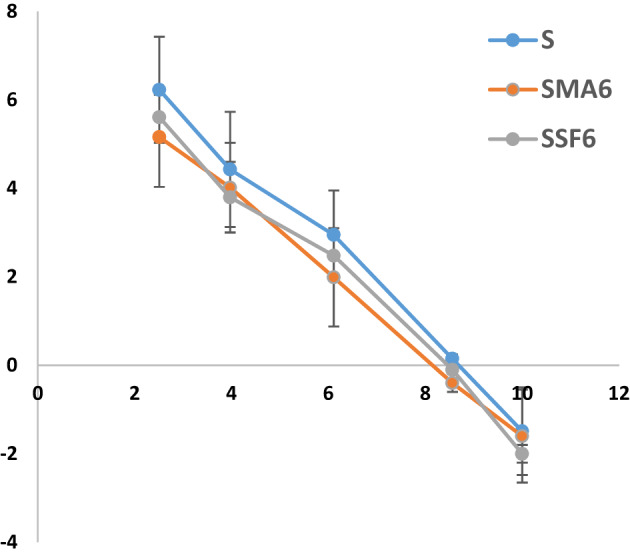
The point of zero charge of various prepared geopolymer composites.

**Fig. 10:**
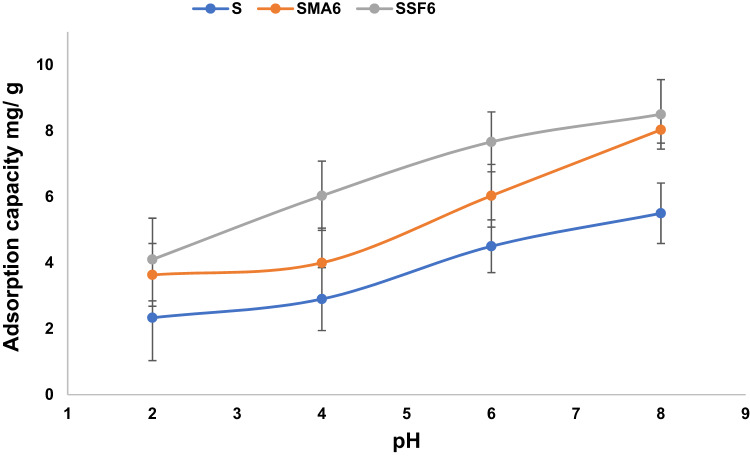
Effect of pH on CV adsorption capacity (Time 60 min, weight of adsorbent 0.05 g, concentration of dye 10 mg/L).

The point of zero charge (pHpzc) is crucial for understanding how geopolymer adsorbents interact with crystal violet (CV) dye^52^. It dictates the geopolymer’s surface charge and, consequently, its ability to remove CV ions. To ensure effective adsorption, a pH range of 2–8 was used. While factors like chemical composition and curing conditions influence the pHpzc, adding MLA and SF had little effect on the pHpzc in the studied geopolymers, which was estimated to be around 8 as shown in Fig. 9 and Table 4 It was observed that the adsorption capacity (Fig. 10) and removal efficiency (Table 5) increase as the pH increases up to 8Consequently, the geopolymer surface becomes positively charged when the pH of the solution falls below the pHpzc, which results in electrostatic repulsion with the positively charged CV ions and decreased adsorption^53,54^. Conversely, at pH values close to the pHpzc, the surface gains a negative charge, which significantly enhances electrostatic attraction and thus improves adsorption performance. This finding allies with the results of other investigations that have been published in the literature^55–60^.

**Table 4:** The point of zero charge of various prepared geopolymer composites.

Geopolymer mix	S	SMA6	SSF6
PZC	8.70	8.20	8.50

**Table 5:** Removal efficiency values of various prepared geopolymer composites.

		S	SMA6	SSF6
pH	2	24.63	36.20	40.20
	4	29.11	40.11	60.30
	6	45.60	60.11	77.60
	8	57.42	77.07	85.98
Dose	0.01	58.59	74.31	77.52
	0.05	65.42	77.07	85.98
	0.07	69.35	78.14	89.66
	0.1	68.92	79.22	91.21
	0.15	72.10	82.54	93.59
	0.2	73.23	84.68	95.05
Time (min.)	30	52.69	74.32	77.73
	60	50.59	74.31	77.52
	120	46.95	74.14	75.15
	180	46.50	73.31	74.52
	240	46.01	72.31	74.15
	300	45.95	72.14	73.51
	360	45.95	71.31	73.15
Concentration (mg/l)	10	52.69	77.62	77.73
	30	38.14	67.73	70.73
	60	29.17	62.00	64.00
	80	25.56	63.43	63.73
	100	22.00	58.83	62.13
	120	20.90	57.53	60.73

#### Effect of adsorbent dose

**Fig. 11:**
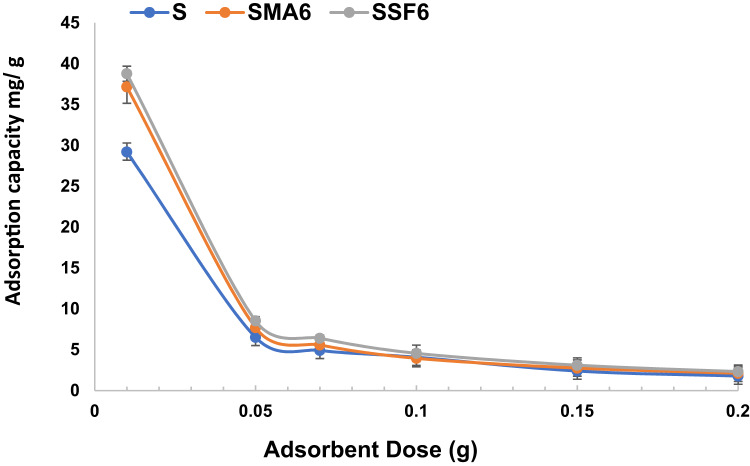
Effect of adsorbent weight on CV adsorption capacity (Time 60 min, pH 8, concentration of dye 10 mg/L).

The amount of adsorbent used has a significant impact on the material’s adsorbent capacity. To improve adsorption performance, the adsorbent dosage was optimized, as shown in Fig. 11. Overall, the adsorption efficiency increases as the dosage of geopolymer composite increases from 0.01 g to 0.2 g (see Table 5). This is because higher doses increase the number of adsorption-active sites, increasing the probability of crystal violet interacting with the geopolymer material. However, the adsorption capacity decreases with doses above 0.01 g, as the adsorbent particles aggregate and the adsorption sites become saturated, reducing the available surface area for adsorption^59^. The following studies were conducted using the ideal adsorbent dosage of 0.01 g.

#### Effect of contact time

**Fig. 12:**
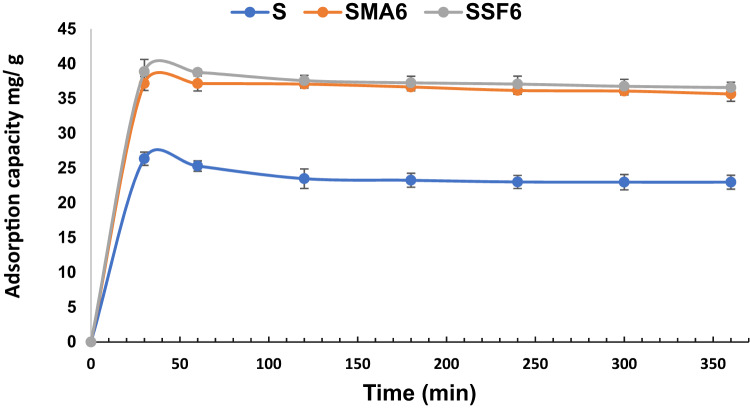
Effect of time on CV adsorption capacity (pH 8, weight of adsorbent 0.01 g, concentration of dye 10 mg/L).

In this investigation, we investigated how contact time affects the adsorption of CV dye on geopolymer composite samples at room temperature. Our findings, as illustrated in Fig. 12 and Table 5, revealed that the majority of the dye was absorbed during the adsorption’s initial half-hour process across all geopolymer samples, albeit with variations in adsorption capacity. The existence of accessible adsorption sites at the beginning stage is responsible for this quick adsorption. After reaching equilibrium and saturating the accessible sites, no further adsorption occurred^59^. Efficiency decreases as the active sites on the geo-adsorbents get saturated, resulting in a lower adsorption rate due to fewer accessible sites for absorbing new dye ions.

#### Effect of dye concentration

**Fig. 13:**
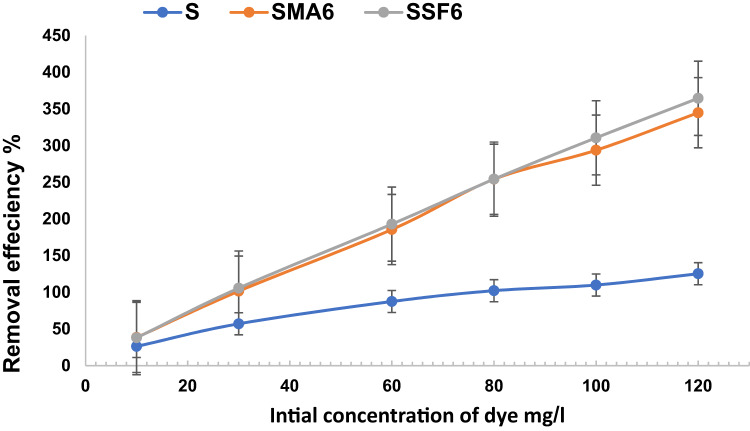
Effect of initial concentration on CV adsorption capacity (Time 60 min, weight of adsorbent 0.01 g, pH8).

The adsorption capacity and removal efficacy at several initial dye concentrations are shown in Fig. 13 and Table 5. Different concentrations of CV were utilized to assess the influence of the initial dye concentration (ranging from 10 mg/L to 120 mg/L) on removal efficiency. The removal efficiency increases alongside the adsorbent’s loading capacity as the initial dye concentration rises, until the number of active sites that can bind with dye molecules is exhausted^52^. A similar study on the effects of these parameters on CV adsorption revealed a similar trend ^52 56^. Based on the result, the adsorption capacity and removal efficiency of Styrofoam slag-based geopolymer composite are higher than moringa leaves ash composite and reference mix, which conforms to the porous structure and higher surface area of SSF6, which is sufficient to adsorb more CV.

#### Adsorption isotherm

**Fig. 14:**
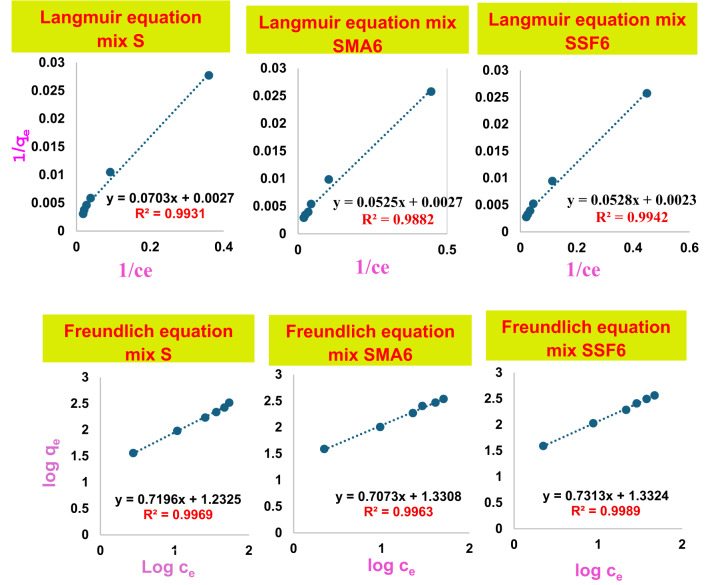
The fitting results of Langmuir model and Freundlich model.

An isotherm for adsorption is essential to elucidate how CV interacts with the various prepared geopolymer composites. Freundlich multi-layer and Langmuir single-layer models were employed to model the equilibrium data for CV adsorption. The Langmuir adsorption model effectively describes single-layer adsorption and is predicated Based on the idea that adsorption takes place on a uniform surface of the adsorbent. Furthermore, adsorbed molecules remain stationary, and all surface sites are equivalent^59,60^. The data collected between 10 and 120 ppm were fitted to the Langmuir adsorption model, as illustrated in Fig. 14, with correlation coefficients (R²) of 0.993, 0.9882, and 0.9989 for S, SMA6, and SFF6, respectively. Table 6 shows K_l_, R_l_, and q_max_ for the geopolymer mixes. The geopolymer’s adsorption of CV has R_l_ values of (0.656, 0.664, and 0.696) for S, SMA6, and SFF6, respectively, indicating a favorable adsorption process. The q_max_ (434.782 mg/g) confirms the improved adsorption performance of SSF. The equilibrium data were subsequently considered utilizing the Freundlich isotherm model, which demonstrated a mechanism of heterogeneous adsorption by plotting log q_e_ vs. log C_e_, as shown in Fig. 14. The Freundlich multi-layer adsorption model describes heterogeneous systems with non-uniform adsorption sites, in contrast to the Langmuir isotherm. The correlation coefficient value (R² = 0.996, 0.9963,0.9942 for S, SMA6, and SFF6, respectively) of the Freundlich model best matched the experimental results of CV adsorption on the geopolymer composite. Consistent with the outcomes regarding the R_l_ value, the results indicated that n has a value greater than unity, suggesting that the adsorption process of CV dye is advantageous for the produced geopolymer. A high value of the Freundlich constant (k_f_) implies that CV can be readily absorbed from an aqueous solution, as shown in Table 6. The results demonstrated that this adsorption interaction process supported the Freundlich theory for geopolymer mixes.

**Table 6:** Isotherm parameters for the adsorption behavior of various modified geopolymer cement mixes towards crystal Violet dye.

Isotherm parameter				
Type of isotherm	Parameters	S	SMA6	SSF6
Langmuir	q_max_ (mg/g)	131.58	370.37	434.78
	k_L_ (L/mg)	0.05	0.05	0.04
	R_L_	0.66	0.66	0.70
	R^2^	0.993	0.9882	0.9989
Freundlich	K_F_	12.20	21.42	21.50
	n	0.52	1.41	1.37
	R^2^	0.996	0.9963	0.9942

#### Adsorption kinetics

**Fig. 15:**
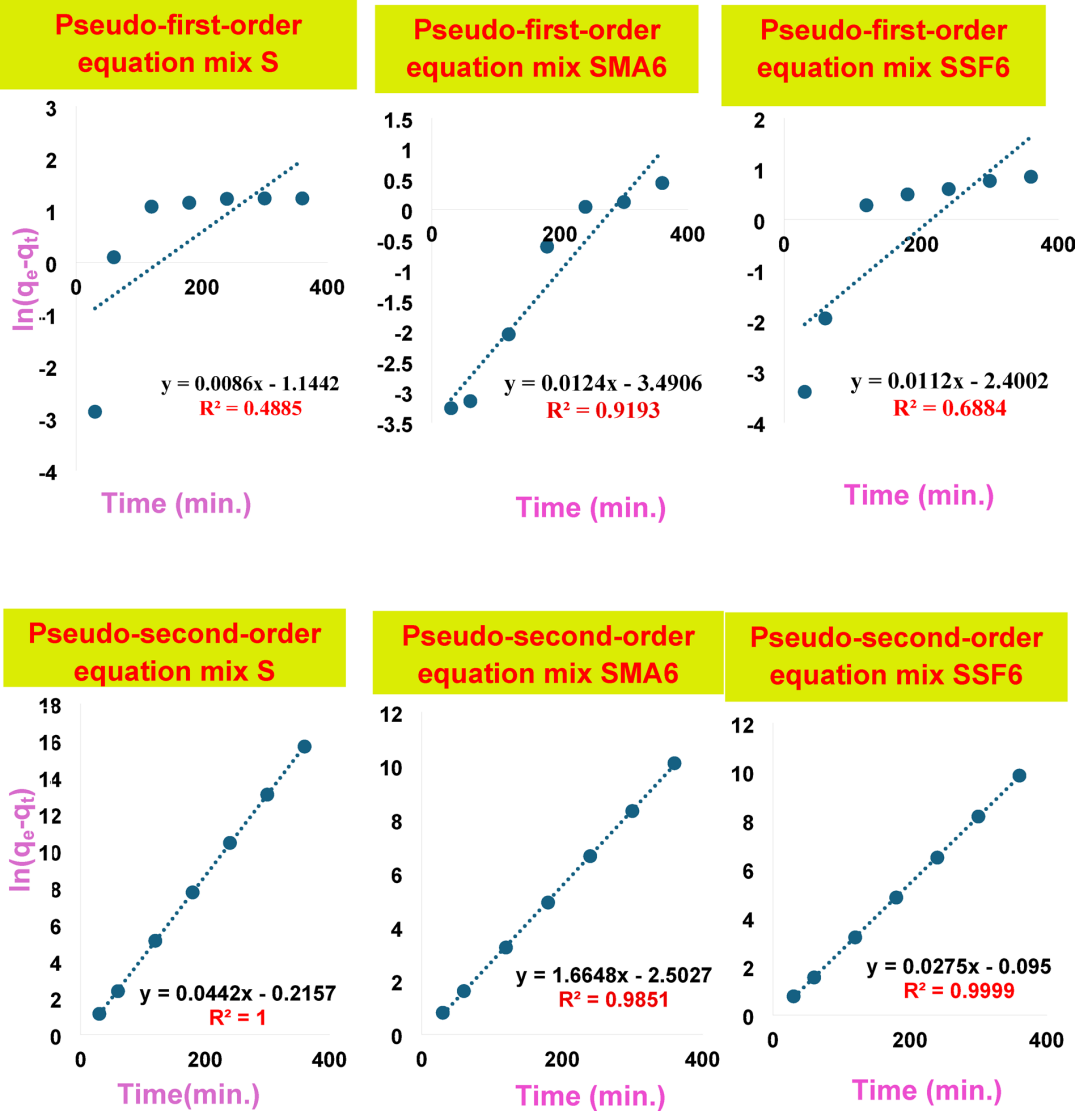
The fitting results of the pseudo-first-order and pseudo-second-order equations.

It is crucial to understand the process and rate of CV adsorption onto geopolymer composites, as the system’s kinetics significantly influences the removal performance. The correlation coefficients and kinetic parameters for CV’s adsorption onto geopolymer composites are illustrated in Fig. 15; Table 7. The correlation coefficient (R^2^) was used to show the consistency of the model and data. Findings showed that the pseudo-first-order kinetic model has a significantly lower correlation coefficient than the pseudo-second-order kinetic model. Additionally, the qe value obtained from the fitted linear plot of the pseudo-second-order model aligns more closely with the experimental data in contrast to the pseudo-first-order model. These findings demonstrated that the adsorption is irreversible and that the rate-determining phase is accomplished by chemisorption^61,62^. It can be attributed to the surface complexation reaction between the negative charges on the geopolymer created by the deprotonation of silanols (SiO–H) and aluminol (AlO–H) groups present and the CV (cationic species) that predominate in a water-soluble solution at the used pH = 8^56^. Comparable kinetic results were reported for the adsorption of CV onto different adsorbent^55,56,62^. In summary, the great fit of the pseudo-second-order model indicates the main element controlling the adsorption of CV is chemical interactions onto geopolymer materials. Multiple phases are also included in the adsorption mechanism, and the increased porosity of SSF geopolymers greatly enhances intra-particle diffusion.

**Table 7:** Kinetic parameters for the adsorption behavior of various modified geopolymer cement mixes towards crystal violet dye.

Kinetic parameter				
Type of model	Parameters	S	SMA6	SSF6
Pseudo-first-order	q_e_ (mg/g)	0.003	0.077	0.090
	K_1_ (L/mg)	0.00007	0.00002	0.00003
	R^2^	0.3625	0.9318	0.6884
Pseudo-second-order	q_e_	22.62	34.01	36.36
	K_2_	−0.005	-0.0076	-0.0081
	R^2^	1	0.9998	0.9999

#### Microstructure of geopolymer composites after the adsorption of crystal violet dye

**Fig. 16:**
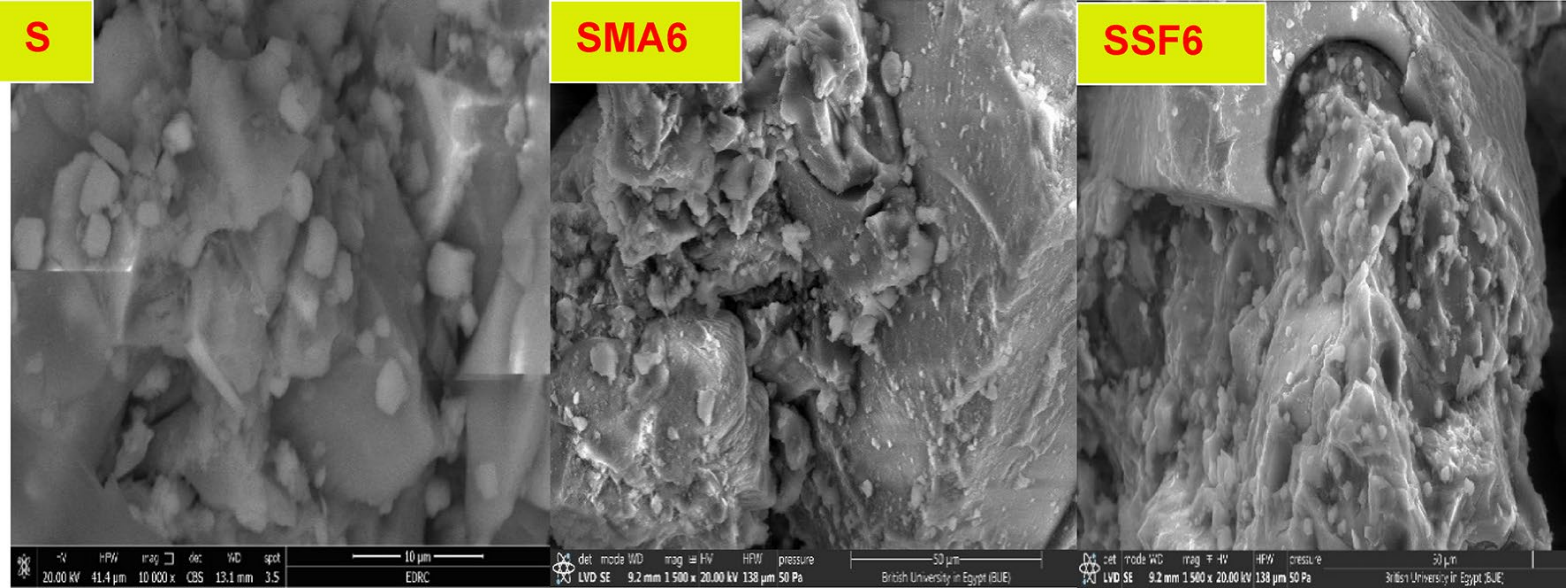
SEM micrographs of geopolymer after adsorption of CV.

The SEM micrographic images of the S, SMA6, and SSF6 after CV adsorption are presented in Fig. 16. It is observed that the surface roughness of the geopolymer particles is somewhat modified, characterized by the adherence of CV to their surfaces. Furthermore, the surface becomes more uniform and less porous as CV molecules fill the cracks, causing them to vanish, indicating that CV dye molecules have been successfully fixed on the surface of the geopolymer^63^.

**Fig. 17:**
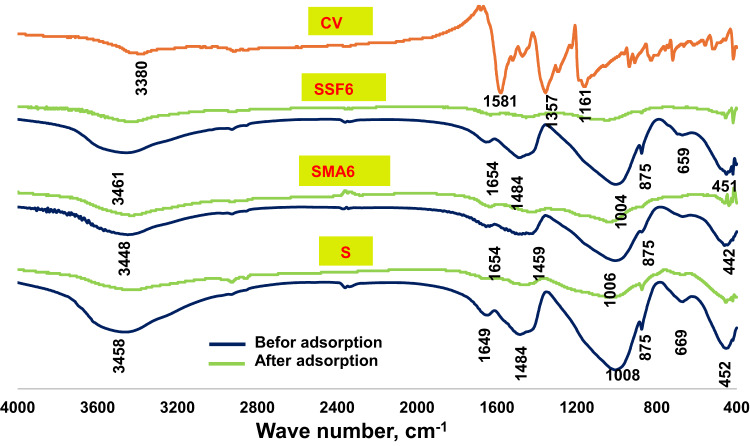
FTIR-spectra of the geopolymer before and after adsorption of CV.

The crystal violet FTIR spectra and the slag geopolymer composites spectra before and upon adsorption are shown in Fig. 17. Crystal violet exhibits bands linked to its functional groups: an aromatic C–H stretching band around 3000 cm^–1^, an aromatic C = C stretching band in the range of 1500–1600 cm^–1^. The band around 2915 cm^–1^ refers to CH_3_-N group characterized by C–N stretching band between 1200 and 1350 cm^–1^^64^. A phenolic O–H stretching band around 3300 cm^–1^^65^. Comparing the bands before and after adsorption, the wide O-H stretching band moved to about 3330 cm⁻¹, which also shows a decrease in intensity, demonstrating the presence of hydrogen bonds between CV and the hydroxyl groups on the geopolymer surface. The reduction in Si–O–T stretching asymmetric vibration bands can also be observed. This suggests that CV may have interacted with the geopolymeric silicate network through electrostatic attraction^66^. The small decrease in minor peaks, like the Si-O/Al-O bending bands, further suggests that the insertion of CV has caused subtle modifications in the framework.

#### Adsorption mechanism of crystal violet on geopolymer composites

The process by which CV dye adheres to geopolymer composites involves a combination of physisorption and chemisorption mechanisms. While the initial dye uptake is largely driven by electrostatic and van der Waals forces, the strong retention of CV molecules is attributed to chemisorption through hydrogen bonding, ionic interactions, and complexation with the geopolymer matrix^67^. Understanding these mechanisms helps in designing more effective adsorbents and optimizing their performance for wastewater treatment applications.

The key factors contributing to physisorption in this system include^68^:

##### Porous structure contribution

Geopolymers typically exhibit micro- and mesoporous structures, which enhance dye uptake by providing a large surface area for adsorption, as confirmed by BET/BJH analyses.

##### Electrostatic interactions

At pH 8, the positively charged CV molecules adhere to the negatively charged geopolymer surface, resulting in strong electrostatic adsorption.

The main chemisorption mechanisms in this system include^67,69^:

##### Hydrogen bonding

Hydrogen bonds are formed between the hydroxyl groups (OH⁻) in the geopolymer structure and the nitrogen atoms (N⁺) in the CV molecules.

##### Ionic interactions

The pseudo-second-order kinetic model suggests that chemisorption plays a dominant role, as electron exchange occurs linking the functional groups of CV and the geopolymer surface.

##### Complexation with active sites

The geopolymer matrix contains Si-O and Al-O functional groups that can participate in electron sharing or exchange interactions, forming stable bonds with dye molecules.

**Fig. 18:**
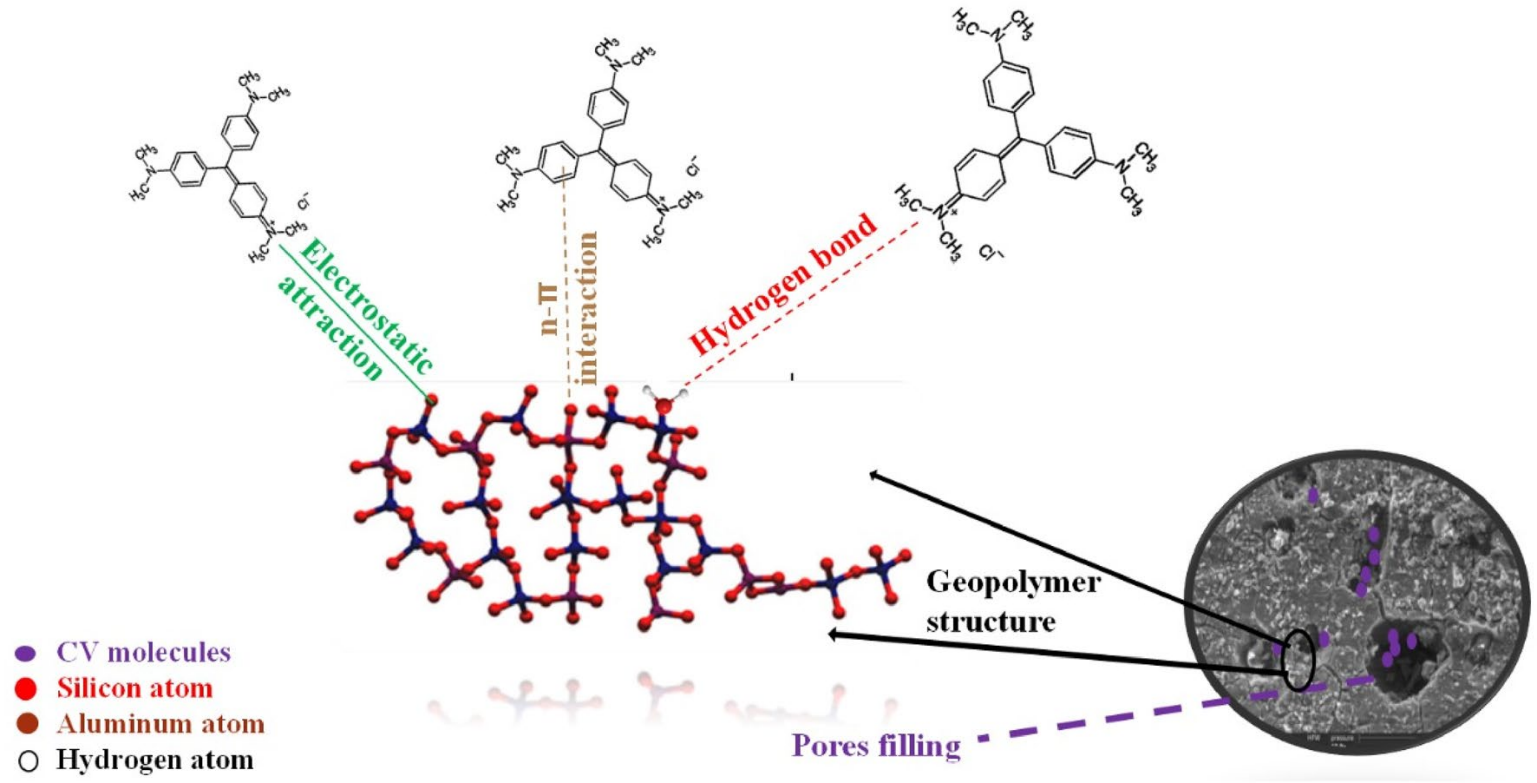
Schematic diagram of the proposed adsorption mechanism.

Figure 18 shows the Schematic diagram of the proposed adsorption mechanism.

#### Comparative analysis with adsorbents from other research studies

Table 8 presents a comparative analysis of the synthesized geopolymers’ crystal violet dye adsorption capacity against other adsorbents documented in prior research. Comparatively, different adsorbents exhibit varying levels of efficiency (Table 8). A maximum capacity of 454.5 mg/g for CV dye is demonstrated by ball milled royal palm leaf sheath powder at a dosage of 40 mg/L, initial concentration of 100 mg/L, contact period of 60 min, pH 6, and 30 °C. However, the minimum adsorption capacity (14.66 mg/g) is given by acid-activated volcanic ash/rice husk based geopolymer at dose of 0.2 g, pH 6, 60 g/l dye concentration and 60 min. While earlier studies have thoroughly examined adsorbents derived from various industrial and agricultural by-products, the geopolymers developed in this study exhibit competitive performance, achieving a maximum adsorption capacity of 434.7 mg/g for SSF6. This enhanced capacity is attributed to the synergistic effect of incorporating Styrofoam (SF) and moringa leaf ash (MLA), which increased the specific surface area of SSF6 and SMA to 53 and 17.50 m^2^/g, thereby providing more active sites for crystal violet adsorption. The improved performance of geopolymers made from Styrofoam and moringa leaf ash positions them as highly effective dye adsorbents. Additionally, these materials contribute to the creation of sustainable construction products by utilizing waste by-products. This use of waste materials not only helps reduce the amount of refuse sent to landfills but also promotes environmental sustainability. The development of these geopolymers showcases the potential for innovative solutions that address both environmental and industrial challenges.

**Table 8:** Comparison between adsorption values for CV by using different adsorbents.

Adsorbent	Form	Maximum capacity (mg/g)	Operational condition					Reference
			Dose	Time	Initial concentration	pH	Temperature	
Banana stem biochar (Pyrolyzed at 350 °C)	Powder	208.33	25 mg	60 min.	100 g/L	3 pH	25 °C	^73^
The acid-activated volcanic ash/rice husk ash-based geopolymer (GPa)	Powder	14.66	0.2 g	60 min.	60 g/L	6 pH	25 °C	^74^
Mesoporous geopolymer using metakaolin and rice husk ash	Powder	276.9	1.5 g/l	120	50 mg/L	4.5 pH	54.85 °C	^75^
Royal palm leaf sheath powder	Powder	344.83	40 mg	60 min.	100 g/L	6 pH	30 °C	^76^
Ball milled Royal palm leaf sheath powder	Powder	454.5	40 mg	60 min.	100 g/L	6 pH	30 °C	^76^
Optimized geopolymer (GPO)	Powder	69.19	1.5 g/L	120	20–140 mg/L	6.5 pH	20 °C	^77^
Vermiculite based geopolymer	Powder	29.6	0.3 g	15 min.	30 g/L	8 pH	30 °C	^28^
Moringa seeds powder slag based geopolymer (SM3)	Powder	322.58	0.01 g	30 min.	120 g/L	8 pH	25 °C	^26^
MLA6	Powder	370.370	0.01 g	30 min.	120 g/L	8 pH	25 °C	Present study
SSF6	Powder	434.782	0.01 g	30 min.	120 g/L	8 pH	25 °C	Present study

#### Limitations of our study

Slag-based geopolymer composites incorporating Styrofoam and Moringa Leaf Ash present in this study a promising approach to sustainable wastewater treatment for removing Crystal Violet dye, promoting circular economy principles using agricultural and industrial wastes. However, evaluating the geopolymer’s ability to regenerate and be reused remains a significant challenge. Recovering and and further treatment techniques are needed to regenerate geopolymer materials after dye adsorption, as noted in previous studies^70–72^. The long-term effectiveness of the geopolymer as an adsorbent may be reduced if the dye adsorption process causes structural and chemical changes in the material^70–72^. This highlights the urgent need for effective and affordable regeneration techniques. Current regeneration methods are often complicated, resource-consuming, and may weaken long-term performance^70–72^. Developing efficient and cost-effective regeneration solutions is critical to preserving adsorption capacity and structural integrity through multiple cycles^70–72^. Further research is essential to create such techniques, with careful attention to the environmental impact of the regeneration process to ensure true sustainability.

Our future research will focus on improving the specific surface area of geopolymers and developing effective regeneration methods. By increasing the surface area, we aim to enhance adsorption capacity and dye removal efficiency, possibly using pore-forming agents or optimizing synthesis processes. Additionally, creating robust regeneration techniques is essential. We will explore innovative approaches to reduce the use of harsh chemicals and energy-consuming methods, such as thermal treatment. Our ultimate goal is to develop sustainable regeneration strategies. This will help lower the environmental impact of the process and support the overall sustainability of the dye removal system. By prioritizing both efficiency and eco-friendliness, we intend to establish new standards for regeneration techniques in water treatment.

## Conclusions

This research explores using industrial waste (ground granulated blast-furnace slag and Styrofoam) and agricultural waste (Moringa leaf ash) to create geopolymer cement. The goal is to develop a material with strong mechanical properties and the ability to absorb harmful dyes, like crystal violet, from wastewater. The study produced two geopolymer composites, each incorporating either Styrofoam or Moringa leaf ash, aiming to provide both economic and environmental advantages. This work advances sustainable construction and wastewater treatment solutions.

- The incorporation of MLA and Styrofoam generally reduces compressive strength and increases porosity in geopolymeric composites but still have mechanical integrity.
- Nitrogen adsorption and desorption analyses for the prepared geopolymer composites near to Type IV than Type VI isotherms with H3 hysteresis, indicating the existence of mesoporous structures. Notably, the SSF6 geopolymer composite, characterized by its fine mesoporous structure and the highest surface area, exhibited a significantly enhanced adsorption capacity compared to the control mix (S).
- In adsorption performance, the most successful sample was determined to be the SSF6 geopolymer. The SSF6 geopolymer showed a maximum CV capacity of 434.782 mg/g. under ideal circumstances (pH 8, contact period of 30 min, adsorbent dosage of 0.01 g L^-1^, and beginning dye concentration of 10 mg L^-1^).
- The adsorption mechanism demonstrated multilayer chemisorption by adhering to the Freundlich isotherm and pseudo-second-order kinetic model.
- The adsorption of crystal violet dye onto geopolymer composites is influenced by both physisorption and chemisorption. Initial adsorption is driven by the porous structure of the geopolymer and electrostatic interactions, while stronger retention occurs through hydrogen bonding, ionic interactions, and complexation between the dye and the geopolymer matrix.

From our study findings, incorporating industrial and agricultural waste into geopolymer composites has improved their adsorption behavior. This enhancement increases their effectiveness in environmental applications, particularly in wastewater treatment. Additionally, recycling industrial and agricultural waste to produce new building materials serves a dual purpose, benefiting both environmental sustainability and the construction industry. Future research should concentrate on pilot-scale testing, investigate more diverse pollutant types, and evaluate substitute waste-derived activators in order to improve sustainability and lower manufacturing costs. Large-scale viability should also be assessed using life cycle and techno-economic analyses.

## Data Availability

The authors declare that the data supporting the findings of this study are available within the paper. Should any raw data files be needed in another format, they are available from the corresponding author upon reasonable request. Source data are provided with this paper.

## Abbreviations

CV: Crystal violet
MLA: Moringa leaves ash
SF: Styrofoam
LSS: Liquid sodium silicate
GGBFS: Ground granulated blast furnace slag
CAH: Calcium aluminate hydrate
CSH: Calcium silicate hydrate
CASH: Calcium alumino silicate hydrate
NASH: Sodium ammonium silicate hydrate
XRD: X-ray diffraction
SEM: Scanning electron microscopy
FTIR: Fourier-transform infrared spectroscopy
BET: Brunauer-Emmett-Teller
BJH: Barrett-Joyner-Halenda
pHpzc: The point of zero charge
PC: Portland cement
